# Synthesis and Spectrosopic Identification of Hybrid 3-(Triethoxysilyl)propylamine Phosphine Ruthenium(II) Complexes

**DOI:** 10.3390/molecules15053618

**Published:** 2010-05-17

**Authors:** Ismail Warad, Saud Al-Resayes, Zeid Al-Othman, Salem S. Al-Deyab, El-Refaie Kenawy

**Affiliations:** 1 Department of Chemistry, King Saud University, P. O. Box 2455, Riyadh 11451, Saudi Arabia; 2 Petrochemical Research Chair, Department of Chemistry, King Saud University, P. O. Box 2455, Riyadh 11451, Saudi Arabia

**Keywords:** Ru(II) complexes, phosphine, ether-phosphine, EXAF, Sol-gel, NMR

## Abstract

An investigation into the potential ruthenium(II) **1-3 **complexes of type [RuCl_2_(P)_2_(N)_2_] using triphenylphosphine and 1,3-*bis-*diphenylphosphinepropane and 3-(triethoxysilyl)propylamine has been carried out at room temperature in dichloromethane under an inert atmosphere. The structural behaviors of the phosphine ligands in the desired complexes during synthesis were monitored by ^31^P{^1^H}-NMR. The structure of complexes **1**-**3** described herein has been deduced from elemental analyses, infrared, FAB-MS and ^1^H‑, ^13^C- and ^31^P-NMR spectroscopy. Xerogels **X1-X3** were synthesized by simple sol-gel process of complexes **1**-**3** using tetraethoxysilane as co-condensation agent in methanol/THF/water solution. Due to their lack of solubility, the structures of **X1-X3** were determined by solid state ^13^C-, ^29^Si- and ^31^P-NMR spectroscopy, infrared spectroscopy and EXAFS.

## 1. Introduction

Phosphines and diphosphines have been intensively used as monodentate and bidentate ligands in coordination chemistry because of their electron-donating power [1,2,3,4,5,6,7,8,9,10]. Metal complexes containing phosphorus ligands have always been important, due to their possible catalytic activity, and a variety of them have already been reported in literature [10,11,12,13,14,15,16,17,18,19,20,21,22,23,24,25,26,27,28,29,30,31,32,33,34,35]. In general diphosphine forms more stable complexes than non-chelating phosphine analogues under the harsh reaction conditions required for catalysis [5,6,7,8,9,10,11,12,13,14,15,16,17,18,19,20,21,22,23,24,25,26,27,28,29,30].

Ether-phosphine P~O ligands are designed to act as monodentate (P~O) as well as bidentate (P^∩^O) donor ligands. Due to the hemilabile character of the ether-phosphine ligand, the oxygen donor is regarded as an intramolecular solvent impeding decomposition of the complex by protection of vacant coordination sites [16,17,18,19,20,21,22,23,24]. The weak ruthenium-oxygen bonds in bis(chelate)ruthenium(II) complexes of the type Cl_2_Ru(P^∩^O)_2_ are easily cleaved during the reaction with other incoming ligands such as amine or diamine [20,21,22,23,24]. By employing ether-phosphine ligands in the synthesis of ruthenium(II) complexes, the introduction of diamines is kinetically controlled and the formation of by-products can be avoided [17,18,19,20,21,22] Diaminediphosphineruthenium(II) complexes with ether-phosphine and classical phosphine ligands were already successfully employed in the catalytic hydrogenation of unsaturated ketones with high diastereo- and enantioselectivity [14,15,21,25,26,27,28]. 

Exchange of triphenylphosphine (PPh_3_) or 1,3-*bis-*diphenylphosphinepropane (dppp) ligands on ruthenium(II) complexes by diamine or amine ligands to produce new families of ruthenium(II)/phosphine/amine complexes is currently one of our lines of investigation [1,3,10]. Due to the presence of phosphine atoms in the backbone of the coordinated ligands, the reaction or fluxional behavior of such complexes can be easy monitored by ^31^P{^1^H}-NMR. Due to the sensitivity of the phosphorus atom to the chemical environment considerable efforts have been expended to study the structural and ligand exchange behavior in ruthenium(II) complexes containing ether-phosphines or diphosphine ligands by following the ^31^P{^1^H}-NMR chemical shift changes [1,3,17,18,19,20,21,22,23,24]. 

The immobilization of metal complexes enables the long-term use of expensive or toxic catalysts and provides a clean and straightforward separation of the product(s) [36]. Compared to organic polymers, inorganic material-immobilized catalysts possess some advantages [37]. For example, they prevent the intermolecular aggregation of the active species because of their rigid structures, they do not swell or dissolve in organic solvents, and often exhibit superior thermal and mechanical stability under the catalytic conditions.

A typical interphase is generated by simultaneous co-condensation of T-functionalized ligands with various alkoxysilanes [1]. By the introduction of triethoxysilyl function group into the amine ligands coordinate complexes, these complexes can be easily supported to a polysiloxane matrix by sol-gel process in order to immobilize catalysts [1,30,31,32,33,34]. 

In this work a set of complexes of general formula RuCl_2_(P)_2_(N)_2_ were prepared using monodentate phosphine and chelate diphosphine ligand in the presence of the monodentate 3-(triethoxysilyl)-propylamine co-ligand. The presence of Si(OEt)_3 _anchoring groups enabled the immobilization of the ruthenium(II) complexes through a simple sol-gel process using Si(OEt)_4_ as cross-linker. 

## 2. Results and Discussion

### 2.1. Synthesis and ^31^P-NMR investigation of ruthenium(II) complexes ***1-3*** and xerogels ***X1-X3***

**Scheme 1:**
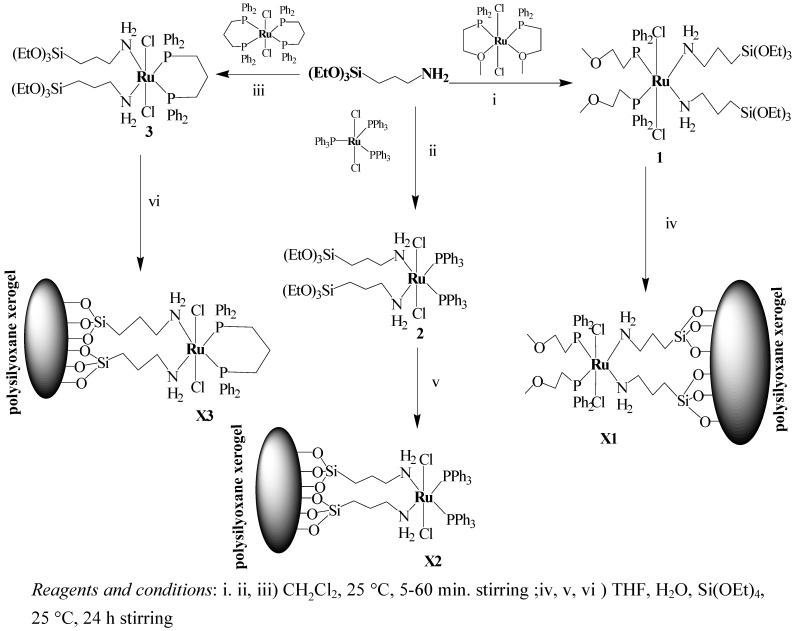
The synthetic route to prepare **1-3** complexes and **X1-X3** xerogels.

Three neutral Ru(II) complexes with PPh_3_, 2-(diphenylphosphino)ethyl methyl ether (ether-phosphine, P~O), dppp ligands were coordinated with monodentate amine ligand in order to produce complexes of the *trans*Cl_2_Ru(P)_2_(N)_2 _type. Treating each of Cl_2_Ru(P^∩^O)_2_, Cl_2_Ru(PPh_3_)_3_ and Cl_2_Ru(dppp)_2_ with two equivalent of 3-(triethoxysilyl)propylamine in dichloromethane resulted in the formation of complexes **1-3**, respectively, as shown in Scheme 1. Yellow powders with high melting points were obtained in very good yields. These complexes are soluble in chlorinated solvents such as chloroform, dichloromethane and insoluble in polar or non-polar solvents like water, methanol, diethyl ether and *n*-hexane. 

Treating of Cl_2_Ru(P^∩^O)_2_ with a slight excess of two equivalents of 3-(triethoxysilyl)propylamine in dichloromethane produced complex **1 **as the *trans*-Cl_2_Ru(P~O)_2_(NH_2_R)_2 _isomer in very good yield. The stepwise formation was monitored by ^31^P{^1^H} spectroscopy, in the NMR tube experiment, addition of 3-(triethoxysilyl)propylamine to CDCl_3_ solution containing Cl_2_Ru(P^∩^O)_2_ generated a high field shift from δ_p _= 64.4 ppm to δ_p _= 40.8 ppm.

**Figure 1:**
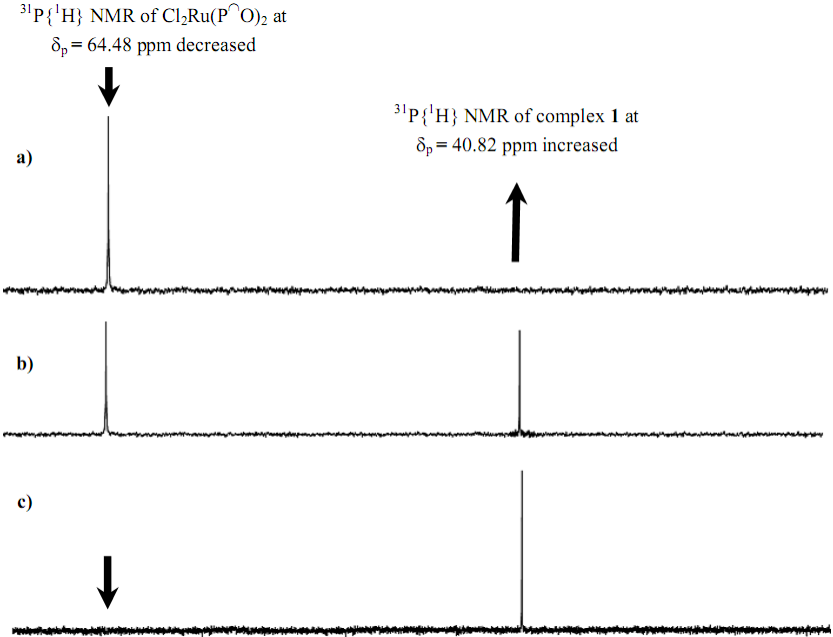
Time-dependent ^31^P{^1^H}-NMR spectroscopic of Cl_2_Ru(P^∩^O)_2 _at δ_p _= 64.4 ppm mixed with two equivalent of 3-(triethoxysilyl)propylamine co-ligand in CDCl_3_ in the NMR tube to produce complex 1 at δ_p _= 40.8 ppm a) before co-ligand addition, b) 1 min. and c) 2 min. after the co-ligand addition.

The immediate disappearance of the complex Cl_2_Ru(P^∩^O)_2_ signal at δ_p _= 64.4 ppm upon 3-(triethoxysilyl)propylamine addition in parallel to the appearance of another signal at δ_p _= 40.8 ppm is related to formation of complex **1**, which was completed in two minutes without side product formation as shown in Figure 1.

The two weak Ru-O bonds in Cl_2_Ru(P^∩^O)_2 _were cleaved by the two incoming molecules of the 3-(triethoxysilyl)propylamine co-ligand to form the two new Ru-N bonds of complex **1**. The presence of the hemilabile ether-phosphine ligand accelerated and stabilized such synthesis without any side products. 

In the preparation of complex **2**, one molecule of PPh_3 _ligand in Cl_2_Ru(PPh_3_)_3_ was exchanged quantitatively by two equivalents of 3-(triethoxysilyl)propylamine in dichloromethane to form the most stable 18 electron valance shell complex **2** at δ_p _**=** 45.8 ppm as *trans*-Cl_2_Ru(dppp)(NH_2_R)_2 _in good yield.

**Figure 2:**
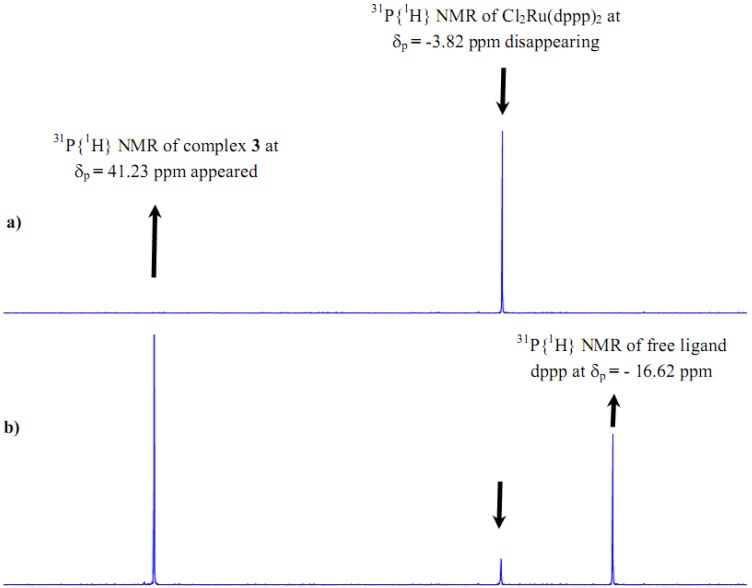
Time-dependent ^31^P{^1^H}-NMR spectroscopic of Cl_2_Ru(dppp)_2 _at δ_p _= -3.82 ppm mixed with two equivalent of 3-(triethoxysilyl)propylamine co-ligand in dichloromethane to produce complex 3 at δ_p _= 41.23 ppm a) before ligand addition, b) 20 min. after ligand addition.

Complex **3 **was obtained by a substitution reaction starting from Cl_2_Ru(dppp)_2_ treated with 3-(triethoxysilyl)propylamine. Mixing of Cl_2_Ru(dppp)_2_ with a slightly excess of two equivalents of 3-(triethoxysilyl)propylamine in dichloromethane enabled the preparation of complex **3** in a very good yield. The stepwise formation of complex **3 **is easily monitored by ^31^P{^1^H} spectroscopy. Addition of 3-(trimethoxysilyl)propylamine in dichloromethane solution containing Cl_2_Ru(dppp)_2_ generates a downfield shift of ~ 45 ppm. One molecule of dppp ligand is exchanged rapidly by two molecules of the 3-(triethoxysilyl)propylamine co-ligands within 20 min to produce a complex of the *trans*-Cl_2_Ru(dppp)(NH_2_R)_2 _type, traces of Cl_2_Ru(dppp)_2_ at δ_p_ = -3.8 ppm, in addition to the free dppp at δ_p _= -16.6 ppm and the product complex **3** at δ_p _= 41.2 ppm were recorded, as shown in Figure 2.

In these reactions, the combination of the change in the color from brown to light yellow and the ^31^P{^1^H}-NMR data confirmed the [1:2] diphosphine:amine fast-exchange reaction without any unexpected side reactions.

Compounds **1-3** were subjected to a sol-gel process with 10 equivalents of Si(OEt)_4_ using methanol/THF/water sol-gel conditions which allowed the preparation of non-soluble polysiloxane xerogels **X1-X3**, respectively. A typical sol-gel polymerization process at room temperature was carried out due to the presence of triethoxysilyl in the backbone of 3-(triethoxysilyl)propylamine ligand and Si(OEt)_4_. THF served as solvent for complexes **1-3**, while alcohol is necessary to homogenize the product and reactant mixture during the sol-gel process and water acts as initiator for the sol-gel process. Due to poor solubility of the **X1-X3** xerogels they were subjected to available solid state measurements like NMR, IR and EXAF. 

**Figure 3:**
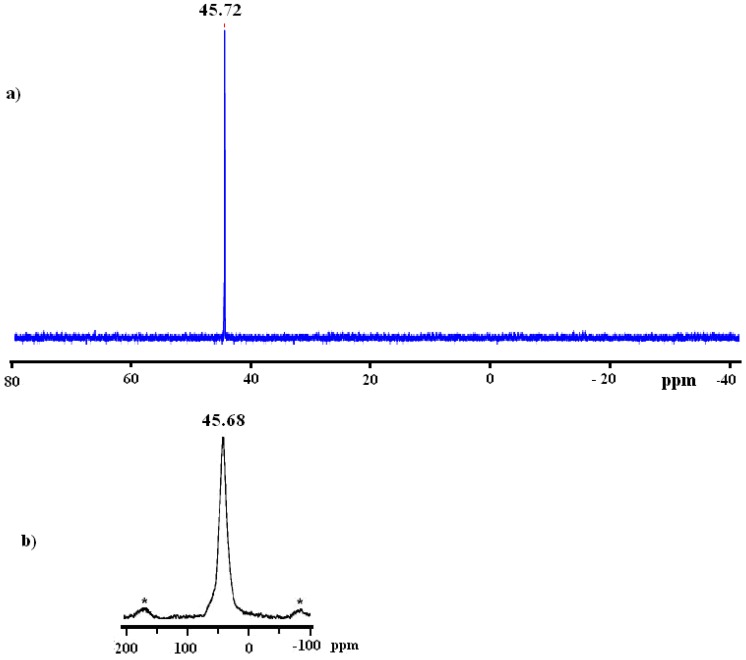
a)^ 31^P{^1^H} of complex 2 in CD_2_Cl_2 _before sol-gel b) ^31^P-CP/MAS-NMR spectrum of **X2** xerogel after sol-gel.

Comparison of the solid state ^31^P-MAS-NMR spectrum of the xerogel **X2 **with the solution phase ^31^P-NMR spectrum of complex **2** corroborated that no significant change of geometry in the coordination sphere of the phosphorus atoms had taken place before or after the sol-gel process, as shown in Figure 3. 

In the spectrum of the solid state material, side bands were observed due to the rotational frequency of the sample during measurements, and the peak was broader in comparison with the solution ^31^P{^1^H}-NMR result for complex **2**. Together the signals and the ^31^P-NMR chemical shifts confirmed that the expected **2** and **X2** complexes were established with identical structure around the ruthenium(II) center atom.

Of interest is the use of ^31^P{^1^H}-NMR as a power tool to gain structural conformation about phosphorous-containing molecules and reaction processes. ^31^P{^1^H}-NMR chemical shifts, integrations, broadness and splitting can provide informative data about the favored isomers in the formation of RuCl_2_(P)_2_(N)_2_ complexes. 

**Scheme 2:**
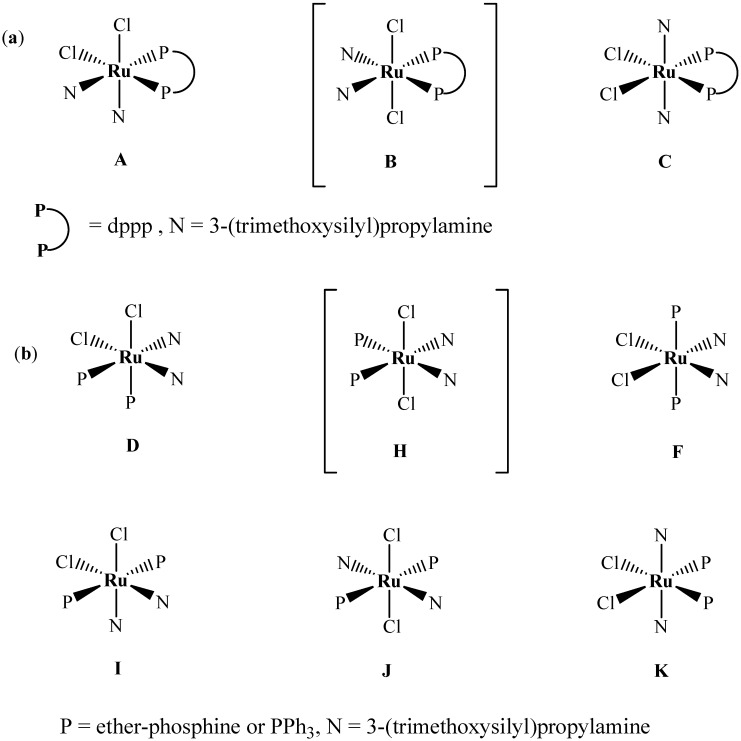
The possible geometries of: (a) three expected isomers of RuCl_2_(PP)(N)_2_ formula N-donor is monodentate amine ligands and PP-donor is bidentate phosphine ligand (dppp), (b) six expected isomers of RuCl_2_(P)_2_(N)_2_ formula, where P-donor is monodentate phosphine ligands (PPh_3_ or P~O).

In case where dppp was used to prepare complex **3**, the spectroscopic data are consistent with the coordination of these ligands in a static bidentate fashion which reduced the isomer number to three as in Scheme 2a, while the use of monodetate phosphine (PPh_3_ or P~O) and amine ligands increased the isomer number to six, as in Scheme 2b.

Due to the expected *C_2_v* symmetry in such complexes, it is easily observed that all the above expected isomers **A-K** should show only sharp signals by P-NMR, except the thermodynamic isomers **A** and **D** which usually reveal an AB pattern P-NMR due to the unequivalent P split atoms in the backbone of the complexes (one P trans to N and the other trans Cl). Based on the ^31^P{^1^H}-NMR chemical shifts and our previous study on such complexes [1,2,10,17,18,19,20,21,22,23,24], it was anticipated that the kinetic favored isomers using both diphosphine and phosphine ligands of type **B** and **H** isomers (*trans*-RuCl_2_ with nitrogen atoms are *trans* to phosphorus atoms) would be structurally favored over any other expected isomers [2,10,17,18,19,20,21,22,23,24]. The kinetically favored products *trans*-[RuCl_2_P_2_N_2_] isomer **B** and **H** were seen at δ_p_ ~ 40, 45, 41 ppm for complexes **1-3** as well as **X1-X2** xerogels, respectively. No traces of the other non-favored isomers were detected by ^31^P{^1^H}-NMR at room temperature using dichloromethane or CDCl_3_ as solvents.

### 2.2. H and C NMR investigations

**Figure 4:**
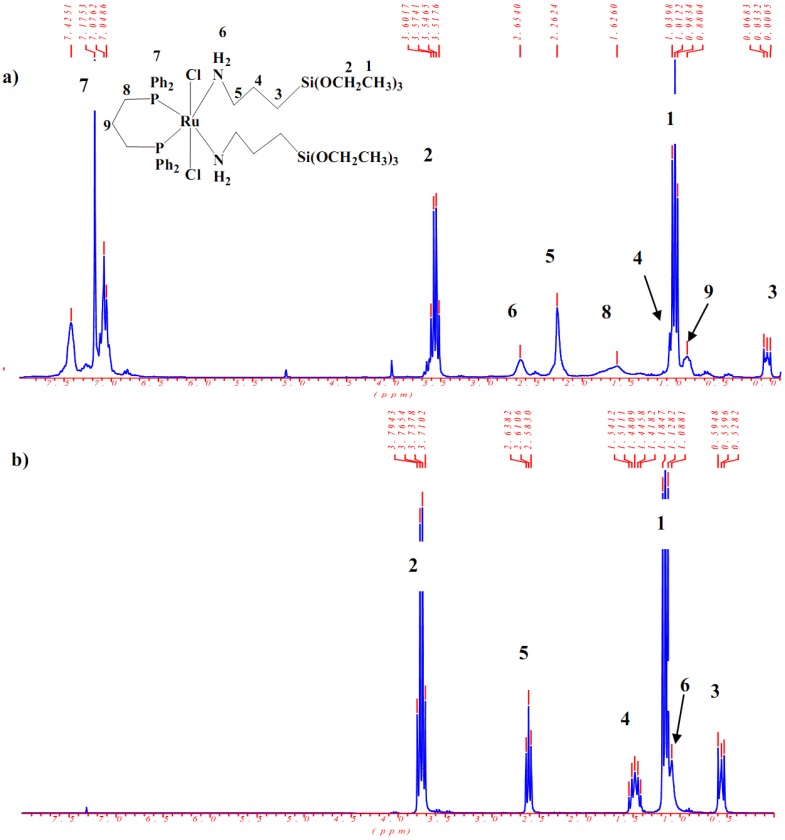
^1^H-NMR of complex **3:** a) and free ligand 3-(triethoxysilyl)propylamine; b) in CDCl_3_ at room temperature.

In the ^1^H-NMR spectra of the amine(phosphine)ruthenium(II) complexes **1-3** characteristic sets of signals were observed, which are attributed to the phosphine as well as 3-(triethoxysilyl)propylamine co-ligands. Their assignment was supported by the free ligand ^1^H-NMR study. The integration of the ^1^H resonances confirmed that the phosphines to amine ratios are in an agreement with the compositions of the desired complexes. As a typical example the ^1^H-NMR of complex **3** was compared by the free 3-(triethoxysilyl)propylamine in Figure 4. 

All the signals of 3-(triethoxysilyl)propylamine co-ligand in complex **3** are shifted to slightly higher field compared to the free ligands, except the H_2_N protons which were moved to lower field from ~1 ppm to 2.6 ppm due to the direct coordination of the nitrogen atom to the ruthenium center. All other protons are in their expected regions, as shown in Figure 4.

**Figure 5:**
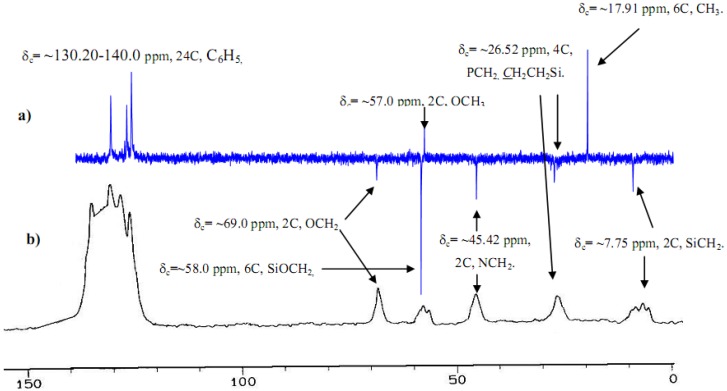
^1^ Dept 135 ^13^C-NMR of complex **1** in CDCl_3_ a) compared by solid state ^13^C-CP-MAS-NMR **X1** xerogels b).

Characteristic sets of resonances phosphine as well as 3-(triethoxysilyl)propylamine are found in the ^13^C{^1^H}-NMR spectra of the desired complexes**, **which are attributed to the aliphatic part of the phosphine and diamine ligands, respectively. AXX´ splitting patterns were observed for the aliphatic and aromatic carbon atoms directly attached to phosphorus. They are caused by the interaction of the magnetically inequivalent phosphorus atoms with the ^13^C nuclei. This pattern is also consistent with isomers **B** and **H** (Scheme 2). Examination of the ^13^C-CP-MAS-NMR spectrum of the modified solids along with the solution phase spectrum of the corresponding molecular precursor led to the conclusion that the organic fragments in **1 **and**X1 **remained intact during the grafting and subsequent workup without measurable decomposition (Figure 5). 

The absence of CH_2_O at δ_C_ = 50.8 and CH_3_ at δ_C_ = 18.2 ppm belong to (CH_3_CH_2_O)_3_Si of the 3-(triethoxysilyl)propylamine ligand after the sol-gel process of complex **1** to establish xerogel **X1**, were the major differences noted between spectra, which supported the immobilization of the desired hybrid Ru(II) complexes. The total disappearance of groups in **X1** (Figure 5b), compared by **1** (Figure 5a), provides good confirmation of a sol-gel process gone to full completion.

Solid-state ^29^Si-NMR provided further information about the silicon environment and the degree of functionalization [1,30]. In all cases, the organometallic/organic fragment of the precursor molecule was covalently grafted onto the solid, and the precursors were, in general, attached to the surface of the polysiloxane by multiple siloxane bridges. The presence of T^m^ sites in case of xerogel **1-3** (with m = 2 and 3) in the spectral region of T^2^ at δ_Si_ = -57.8 ppm and T^3^ at δ_Si_ = -67.1 ppm as expected, Q silicon sites due to Si(EtO)_4_ condensation agent were also recorded to Q^4^ at δ_Si_ = -109.5 ppm silicon sites of the silica framework.

### 2.3. IR investigations

**Figure 6:**
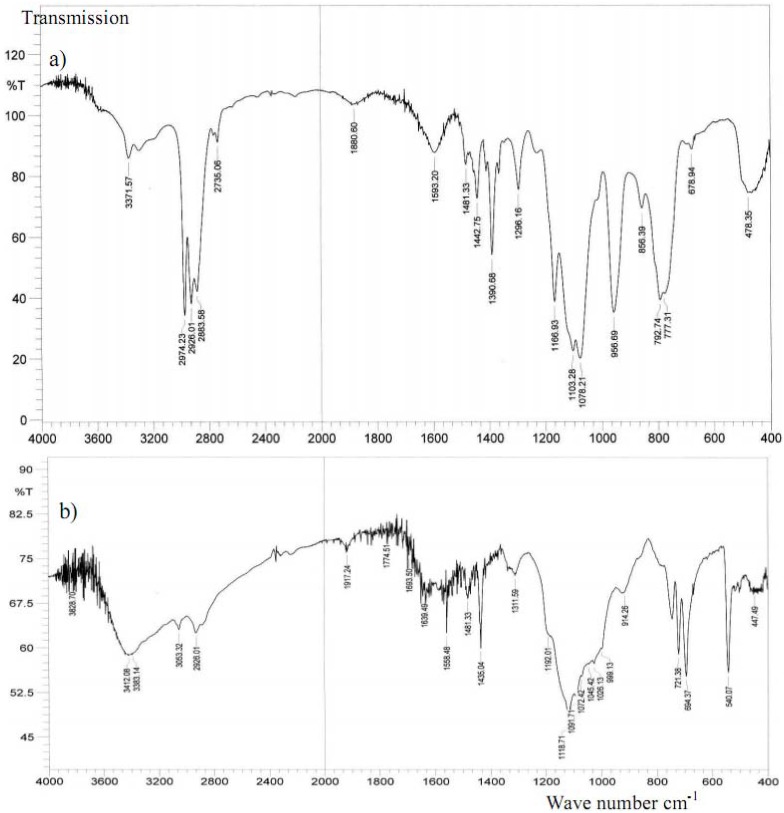
Infra-red spectra (a and b) of **3** and **X3**, before and after sol-gel, respectively.

In order to study the binding mode of the 3-(triethoxysilyl)propylamine and phosphine ligands to the ruthenium complexes and IR study was undertaken. The IR spectra of the desired complexes in particular show several peaks which are attributed to stretching vibrations of the main functional groups in the 3,490–3,300 cm^-1^ (*v*_NH_), 3,280–3,010 cm^-1^ (*v*_PhH_) and 3,090–2,740 cm^-1 ^(*v*_CH_) ranges. All other characteristic bands due to the other function groups are also present in the expected regions. To compare the structural vibration behaviors of these compounds against the infrared spectra of **3** and **X3 **before and after the sol-gel processes are illustrated as typical examples in Figure 6.

The broad intensive stretching vibrations at 2980–2840 cm^-1^ and bending vibration at 1000–950 cm^‑1^ belonging to (*v*_CH_) of the SiOCH_2_CH_3_ function groups of complex **3** as in Figure 6a totally disappeared after the sol-gel process to prepare complex **X3** as seen in Figure 6b, which strongly confirms the completion of the sol-gel process formation. 

### 2.4. EXAFS measurement of *Cl_2_Ru(dppp)_2_ complex and* xerogel ***X3***

EXAFS of starting material Cl_2_Ru(dppp)_2_ complexes was measured before 3-(triethoxysilyl)-propylamine addition then compared by EXAF of **X3 **after sol-gel process of complex **3** to support the ligand exchange method of synthesis as well as to determine the bond lengths between the metal center and the coordinating atoms of the ligands. 

**Figure 7:**
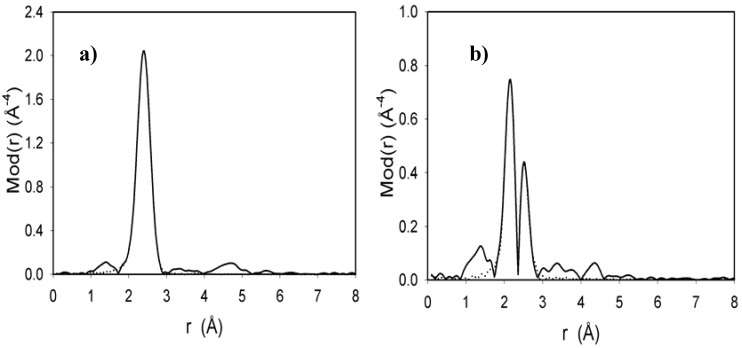
Experimental (solid line) and theoretical (dotted line) Fourier Transform plot of Cl_2_Ru(dppp)_2_ (a) and xerogel **X3** (b) measured at Ru K-edge.

The k^3^ weighted EXAFS function of Cl_2_Ru(dppp)_2_ can be best described by six different atom shells, four equivalent phosphorus and two chlorine atoms with Ru-P and Ru-Cl bond distances of 2.26 and 2.41 Å, are masked (due to the close in the bond lengths) in one relatively broad peak as in Figure 7a. The k^3^ weighted EXAFS function of **X3** can also be described by six different atom shells. For the most intense peak of the Fourier Transform, two equivalent phosphorus, two nitrogen atoms and two chlorine atoms with Ru-P, Ru-N and Ru-Cl bond distances of 2.26, 2.19 and 2.41 Å, respectively, Ru-P and Ru-Cl bonds also masked in one peak but Ru-N bonds was appeared as an addition single peak compared with EXAF of Cl_2_Ru(dppp)_2_ starting material as in Figure 7b, which strongly support the notion that one dppp ligand was exchanged by two 3-(triethoxysilyl)propylamine as well as no change around the ruthenium(II) between **3** and **X3** was detected due to the sol-gel immobilization. 

## 3. Experimental

### 3.1. General remarks, materials, and instrumentation

All reactions were carried out in an inert atmosphere (argon) by using standard high vacuum and Schlenk-line techniques, unless otherwise noted. Prior to use CH_2_Cl_2_, *n*-hexane, and Et_2_O were distilled from CaH_2_, LiAlH_4_, and from sodium/benzophenone, respectively. 1,3–Bis(diphenyl-phosphino)propane (dppp), Cl_2_Ru(P^∩^O)_2_, Cl_2_Ru(PPh_3_)_3_ and Cl_2_Ru(dppp)_2_ were prepared according to literature methods [10,17]. 3-(Triethoxysilyl)propylamine was purchased from Acros. Elemental analyses were carried out on an Elementar Vario EL analyzer. High-resolution liquid ^1^H-, ^13^C{^1^H}-, DEPT 135, and ^31^P{^1^H}-NMR spectra were recorded on a Bruker DRX 250 spectrometer at 298 K. Frequencies are as follows: ^1^H-NMR: 250.12 MHz, ^13^C{^1^H}-NMR: 62.9 MHz, and ^31^P{^1^H}-NMR 101.25 MHz. Chemical shifts in the ^1^H- and ^13^C{^1^H}- NMR spectra were measured relative to partially deuterated solvent peaks which are reported relative to TMS. ^31^P chemical shifts were measured relative to 85% H_3_PO_4_. CP/MAS solid-state NMR spectra were recorded on Bruker DSX 200 (4.7 T) and Bruker ASX 300 (7.05 T) multinuclear spectrometers equipped with wide-bore magnets. Magic angel spinning was applied at 4 kHz (^29^Si) and 10 kHz (^13^C, ^31^P) using (4 mm ZrO_2_ rotors). Frequencies and standards: ^31^P, 81.961 MHz (4.7 T), 121.442 MHz (7.05 T) [85% H_3_PO_4_, NH_4_H_2_PO_4_ (δ = 0.8) as second standard]; ^13^C, 50.228 MHz (4.7 T), 75.432 MHz (7.05 T) [TMS, carbonyl resonance of glycine (δ = 176.05) as second standard]; ^29^Si, 39.73 MHz (4.7 T), 59.595 MHz (7.05 T, (Q8M8 as second standard). All samples were prepared with exclusion of molecular oxygen. IR data were obtained on a Bruker IFS 48 FT-IR spectrometer. Mass spectra: EI-MS, Finnigan TSQ70 (200 °C) and FAB-MS, Finnigan 711A (8 kV), modified by AMD and reported as mass/charge *(m/z).* The EXAFS measurements were performed at the ruthenium K–edge (22118 eV) at the beam line X1.1 of the Hamburger Synchrotronstrahlungslabor (HASYLAB) at DESY Hamburg, under ambient conditions, energy 4.5 GeV, and initial beam current 120 mA. For harmonic rejection, the second crystal of the Si(311) double crystal monochromator was tilted to 30 %. Data were collected in transmission mode with the ion chambers flushed with argon. The energy was calibrated with a ruthenium metal foil of 20 μm thickness. The samples were prepared of a mixture of the samples and polyethylene.

### 3.2. General procedure for the preparation of the complex ***1-3***

3-(Triethoxysilyl)propylamine (0.10 g, 0.455 mmol, 5% excess) was dissolved in dichloromethane (5 mL) and the solution was added dropwise to a stirred solution of Cl_2_Ru(P^∩^O)_2_, Cl_2_Ru(PPh_3_)_3_ or Cl_2_Ru(dppp)_2_ (0.22 mmol) in dichloromethane (5 mL) within 2 min. The mixture was stirred for ca. 2 h at room temperature while the color changed from brown to yellow. Then the solution was concentrated to about 2 mL volume under reduced pressure. Addition of *n*-hexane (40 mL) caused precipitation of a yellow solid powder, which was filtered (P4). After recrystallization from dichloromethane/ diethyl ether, complexes **1-3** were obtained in analytically pure form in very good yields; m.p. > 340 °C (dec.).

Complex **1**: ^1^H-NMR (CDCl_3_): δ (ppm) 0.03 (m, 2H, CH_2_Si), 1.08 (t, 18H, CH_3_), 1.10 (m, 4H, SiCH_2_C*H_2_*),1.48 (br, 4H, PC*H_2_*), 2.06 (br, 4H, CH_2_O), 2.31 (br, 4H, CH_2_N), 2.55 (br, 4H, NH_2_), 2.79 (s, 6H, OCH_3_), 3.66 (m, 12H, OCH_2_), 7.00–7.70 (m, 20H, C_6_H_5_); ^31^P{^1^H}-NMR (CDCl_3_): δ (ppm) 40.82, s, ^13^C{^1^H}-NMR (CDCl_3_): δ (ppm) 7.75 (s, 2C, CH_2_Si), 17.91 (s, 6C, CH_3_), 26.02 (m, 2C, PCH_2_), 26.82 (s, 2C, *C*H_2_CH_2_Si), 45.42 (s, 2C, NCH_2_), 57.93 (s, 2C, OCH_3_), 58.71 (s, 6C, SiOCH_2_), 69.39 (s, 2C, OCH_2_) 127.20–134.0 (m,24C, C_6_H_5_); FAB – MS; *(m/z)*: 1102.3 (M^+^); Anal. Calc. C, 52.26; H, 7.31; Cl, 6.43; N, 2.54 for C_48_H_80_Cl_2_N_2_O_8_P_2_RuSi_2_: Found C, 52.44; H, 7.02; Cl, 6.43; N, 2.44%.

Complex **2**: ^1^H-NMR (CDCl_3_): δ (ppm) 0.05 (m, 2H, CH_2_Si), 1.06 (t, 18H, CH_3_), 1.11 (m, 4H, SiCH_2_C*H_2_*), 2.32 (br, 4H, CH_2_N), 2.71 (br, 4H, NH_2_), 3.61 (q, 12H, OCH_2_), 7.10–7.80 (m, 30H, C_6_H_5_); ^31^P{^1^H} NMR (CDCl_3_): δ (ppm) 45.7. s,^ 13^C{^1^H}-NMR (CDCl_3_): δ (ppm) 7.82 (s, 2C, CH_2_Si), 17.21 (s, 6C, CH_3_), 24.82 (s, 2C, *C*H_2_CH_2_Si), 43.21 (s, 2C, NCH_2_), 56.61 (s, 6C, OCH_2_), 130.12–135.22 (m,36C, C_6_H_5_); FAB–MS; *(m/z)*: 1138.3 (M^+^); Anal. Calc. C, 56.93; H, 6.72; Cl, 6.22; N, 2.46 for C_54_H_76_Cl_2_N_2_O_6_P_2_RuSi_2_: Found C, 56.54; H, 6.42; Cl, 6.60; N, 2.35%.

Complex **3**: ^1^H-NMR (CDCl_3_): δ (ppm) 0.04 (m, 2H, CH_2_Si), 0.88 (br, 2H, PCH_2_C*H_2_*), 1.01 (t, 18H, CH_3_), 1.04 (m, 4H, SiCH_2_C*H_2_*),1.82 (br, 4H, PC*H_2_*CH_2_), 2.26 (br, 4H, CH_2_N), 2.65 (br, 4H, NH_2_), 3.57 (q, 12H, OCH_2_) 6.90–7.50 (m, 20H, C_6_H_5_); ^31^P{^1^H}-NMR (CDCl_3_): δ (ppm) 41.73, s, ^13^C{^1^H}-NMR (CDCl_3_): δ (ppm) 5.52 (s, 2C, CH_2_Si), 16.61 (s, 6C, CH_3_),17.31 (s, 1C, PCH_2_*C*H_2_), 24.22 (s, 2C, *C*H_2_CH_2_Si), 24.82 (m, 2C, PCH_2_), 43.21 (s, 2C, NCH_2_), 56.61 (s, 6C, OCH_2_), 126.09–132.82 (m, 24C, C_6_H_5_); FAB – MS; *(m/z)*: 1026.2 (M^+^); Anal. Calc. C, 52.62; H, 7.07; Cl, 6.90; N, 2.73 for C_45_H_72_Cl_2_N_2_O_6_P_2_RuSi_2_: Found C, 52.34; H, 7.22; Cl, 6.70; N, 2.65%.

### 3.3. General procedure for sol–gel processing of xerogel ***X1-X3***

Complexes **1-3** (0.100 mmol) and Si(OEt)_4_ (1 mmol,10 equivalents) were mixed together in THF (5 mL). The sol–gel took place when a methanol/water mixture (2 mL, 1:1 v/v) was added to the solution. After 24 h stirring at room temperature, the precipitated gel was washed with toluene and diethyl ether (30 mL of each), and petroleum ether (20 mL). Finally the xerogel was ground and dried under vacuum for 24 h to afford after workup ~ 300 mg of a pale yellow powder were collected.

*Xerogel ***X**1: ^31^P-CP/MAS-NMR: δ = 40.9 ppm; ^13^C-CP/MAS NMR: δ (ppm) 6.71 (m, 2C, CH_2_Si), 27.22 (m, 4C, PCH_2_, *C*H_2_CH_2_Si), 45.85 (br, 2C, NCH_2_), 57.93 (m, 2C, OCH_3_), 70.02 (br, 2C, OCH_2_), 125.00–140.00 (m, 24C, C_6_H_5_);^ 29^Si CP/MAS NMR: δ = –67.1 ppm (T^3^), –57.8 ppm (T^2^), -109.5 ppm (Q^4^).

*Xerogel ***X**2: ^31^P-CP/MAS-NMR: δ = 45.7 ppm; ^13^C-CP/MAS NMR: δ (ppm): δ (ppm) 7.82 (m, 2C, CH_2_Si), 24.82 (br, 2C, *C*H_2_CH_2_Si), 45.21 (br, 2C, NCH_2_), 130.12–140.22 (m, 36C, C_6_H_5_); CP/MAS NMR: δ = –67.1 ppm (T^3^), –57.8 ppm (T^2^), -109.5 ppm (Q^4^).

*Xerogel ***X**3: ^31^P-CP/MAS-NMR: δ = 41.7 ppm; ^13^C-CP/MAS NMR: δ (ppm) 6.62 (m, 2C, CH_2_Si), 17.31 (s, 1C, PCH_2_*C*H_2_), 24.22 (s, 4C, *C*H_2_CH_2_Si, PCH_2_), 43.21 (s, 2C, NCH_2_, 120.09–140.82 (m, 24C, C_6_H_5_); CP/MAS NMR: δ = –67.1 ppm (T^3^), –57.8 ppm (T^2^), -109.5 ppm (Q^4^).

## 4. Conclusions

Six ruthenium(II) complexes of the *trans*-[RuCl_2_(P)_2_(N)_2_] type were prepared using three types of phosphine ligands as well as 3-(triethoxysilyl)propylamine co-ligand. ^31^P{^1^H}-NMR was used to study the structural behavior of these complexes during the synthesis. The formation of the kinetically favored isomers of the desired complexes was confirmed by ^31^P-NMR. The presence of T-silyl functions on the amine co-ligand backbone in complexes **1-3** enables the hybridization of these complexes in order to support them on a polysiloxane matrix through sol-gel processes using tetraethoxysilane as co-condensation agent in methanol/THF/water solution. The structure of complexes **1**-**3** described herein has been deduced from elemental analyses, infrared, FAB-MS and ^1^H-, ^13^C-, H, and ^31^P-NMR spectroscopy. Due to their lack of solubility, the structures of xerogels **X1-X3** were determined by solid state ^13^C-, ^29^Si- and ^31^P-NMR spectroscopy, infrared spectroscopy and EXAFS. 
